# FTY720 (fingolimod) modulates the severity of viral-induced encephalomyelitis and demyelination

**DOI:** 10.1186/s12974-014-0138-y

**Published:** 2014-08-20

**Authors:** Caroline A Blanc, Hugh Rosen, Thomas E Lane

**Affiliations:** Department of Molecular Biology and Biochemistry, University of California, Irvine, California 92697-3900 USA; Department of Chemical Physiology, The Scripps Research Institute, La Jolla, California 92037 USA; Department of Pathology, Division of Microbiology & Immunology, University of Utah School of Medicine, Salt Lake City, Utah 84112 USA

**Keywords:** FTY720, S1P receptor, virus, central nervous system, T lymphocytes, demyelination

## Abstract

**Background:**

FTY720 (fingolimod) is the first oral drug approved by the Food and Drug Administration for treatment of patients with the relapsing-remitting form of the human demyelinating disease multiple sclerosis. Evidence suggests that the therapeutic benefit of FTY720 occurs by preventing the egress of lymphocytes from lymph nodes thereby inhibiting the infiltration of disease-causing lymphocytes into the central nervous system (CNS). We hypothesized that FTY720 treatment would affect lymphocyte migration to the CNS and influence disease severity in a mouse model of viral-induced neurologic disease.

**Methods:**

Mice were infected intracranially with the neurotropic JHM strain of mouse hepatitis virus. Infected animals were treated with increasing doses (1, 3 and 10 mg/kg) of FTY720 and morbidity and mortality recorded. Infiltration of inflammatory virus-specific T cells (tetramer staining) into the CNS of FTY720-treated mice was determined using flow cytometry. The effects of FTY720 treatment on virus-specific T cell proliferation, cytokine production and cytolytic activity were also determined. The severity of neuroinflammation and demyelination in FTY720-treated mice was examined by flow cytometry and histopathologically, respectively, in the spinal cords of the mice.

**Results:**

Administration of FTY720 to JHMV-infected mice resulted in increased clinical disease severity and mortality. These results correlated with impaired ability to control viral replication (*P* < 0.05) within the CNS at days 7 and 14 post-infection, which was associated with diminished accumulation of virus-specific CD4+ and CD8+ T cells (*P* < 0.05) into the CNS. Reduced neuroinflammation in FTY720-treated mice correlated with increased retention of T lymphocytes within draining cervical lymph nodes (*P* < 0.05). Treatment with FTY720 did not affect virus-specific T cell proliferation, expression of IFN-γ, TNF-α or cytolytic activity. FTY720-treated mice exhibited a reduction in the severity of demyelination associated with dampened neuroinflammation.

**Conclusion:**

These findings indicate that FTY720 mutes effective anti-viral immune responses through impacting migration and accumulation of virus-specific T cells within the CNS during acute viral-induced encephalomyelitis. FTY720 treatment reduces the severity of neuroinflammatory-mediated demyelination by restricting the access of disease-causing lymphocytes into the CNS but is not associated with viral recrudescence in this model.

## Background

Multiple sclerosis (MS) is a neurodegenerative inflammatory disease of the central nervous system (CNS), which leads to demyelination and progressive neurological disability [1,2]. FTY720, also called Gilenya/fingolimod, is an oral drug recently approved by the Food and Drug Administration (FDA) for treatment of patients with the relapsing-remitting form of MS [3–8]. FTY720 is an immunomodulatory drug that has shown to reduce both acute relapses but also new lesion formation as well as disability progression and brain volume loss in MS patients [9]. The mechanisms for how FTY720 functions are not yet defined; however, the phosphorylated active form of FTY720 (FTY720P) is a sphingosine-1-phosphate (S1P) receptor modulator that inhibits egress of lymphocytes from lymph nodes [9–11]. It is thought that this leads to a dampening of autoreactive T cells specific for myelin antigens infiltrating into the CNS. Importantly, FTY720, due to its lipophilic nature, penetrates the blood–brain-barrier and readily enters the CNS parenchyma [9]. Furthermore, FTY720P is detected *in situ*, suggesting that it may influence the biology of resident cells of the CNS [9].

FTY720 has been shown to improve disease severity in experimental autoimmune encephalomyelitis (EAE), an autoimmune model of neuroinflammation and demyelination commonly used as a model for MS [12–14]. Indeed, therapeutic administration of FTY720 in EAE models is associated with reduced neuroinflammation and improved motor skills [12–15]. In addition to EAE, viral models of demyelination are also relevant tools for studying the pathogenesis of neuroinflammatory-mediated demyelination. For example, infection of susceptible mice with the neurotropic JHM strain of mouse hepatitis virus (JHMV) results in an acute encephalomyelitis followed by chronic demyelination. Like MS, components of the immune system, such as T cells and macrophages, are important contributors to white matter destruction [16–18]. Moreover, JHMV-infected mice undergoing chronic demyelination show similar clinical and histologic disease profiles compared to MS patients [19–21]. As viruses are considered to be a contributing cause of MS [22–35], JHMV infection of the CNS offers not only an excellent model for studying the immunopathological mechanism driving demyelination in MS patients but also can provide insight into effects of MS therapeutics within the context of viral-induced demyelination. We have evaluated the effects of FTY720 on both host defense and disease progression in JHMV-infected mice. Our findings reveal that FTY720 treatment resulted in increased mortality associated with impaired ability to control viral replication. FTY720 did not alter anti-viral effects of T cells, e.g. cytokine secretion or cytolytic activity, but affected lymphocyte egress from draining cervical lymph nodes and accumulation of virus-specific T cells within the CNS. Therefore, FTY720 treatment mutes effective anti-viral immune responses following infection with a neurotropic virus by dampening trafficking of virus-specific T cells to the CNS. Conversely, administration of FTY720 to JHMV-infected mice reduced the severity of demyelination by limiting infiltration of inflammatory T cells into the CNS.

## Methods

### Virus and mice

Age-matched (5 to 7 weeks) S1P1 eGFP knock-in mice (C57BL/6 background) [36] and C57BL/6 mice were anesthetized with an intra-peritoneal (i.p.) injection of 150 μl of a mixture of ketamine (Western Medical Supply, Arcadia, CA, USA) and xylazine (Phoenix Pharmaceutical, Saint Joseph, MO, USA) in Hank’s balanced salt solution. Mice were injected intra-cranially (i.c.) with 150 plaque forming units (PFU) of JHMV (strain V2.2-1) suspended in 30 μl saline [37]. Clinical severity was assessed using a previously described four-point scoring scale [16]. FTY720 (2-amino-2- [2-(4-octylphenyl) ethyl]-1,3-propanediol, hydrochloride) and FTY720P (2-amino-2[2-(4-octylphenyl) ethyl]-1,3-propanediol, mono dihydrogen phosphate ester) were purchased from Cayman Chemical Co (Ann Arbor, MI, USA). Administration of FTY720 or the vehicle was performed by daily i.p. injections of 100 μl starting at day 5 post-infection (p.i.). For analysis of viral titers, mice were sacrificed at defined time points. One half of each brain was removed as well as spinal cords. These were homogenized and used in a plaque assay performed using DBT mouse astrocytoma cell line [38]. Experiments for all animal studies were reviewed and approved by the University of Utah and the University of California, Irvine Institutional Animal Care and Use Committee.

### Cell isolation and flow cytometry

Immunophenotyping of the cellular infiltrate present within cervical lymph nodes, brains and spinal cords of infected mice was accomplished by homogenizing isolated tissue and generating a single-cell suspension for analysis by flow cytometry as previously described [39–41]. In brief, isolated cells were Fc blocked with anti-CD16/32 1:200. The following antibodies were used for immunophenotyping: APC-conjugated rat anti-mouse B220 for B cells; APC-conjugated rat anti-mouse CD4 for CD4+ T cells; PE-conjugated rat anti-mouse CD8 and APC-conjugated rat anti-mouse CD8 for CD8+ T cells; PE-conjugated rat anti-mouse interferon-gamma (IFN-γ) for intracellular cytokine staining; PE-Cy7-conjugated rat anti-mouse CD45, APC-conjugated rat anti-mouse CD19 and PE-conjugated rat anti-mouse CD138 for antibody secreting cells; M133-147 tetramer-PE for virus specific CD4+ T cells and S510-518 tetramer-PE for virus-specific CD8+ T cells. Cell isolates for IFN-γ intracellular staining were cultured in 200 μl RPMI-1640 supplemented with 10% fetal bovine serum, L-glutamine and penicillin-streptomycin, and stimulated *ex vivo* with the immunodominant CD4 epitope (M133-147) or the immunodominant CD8 (S510-518) and Golgi stop for 6 h followed by intracellular staining [42,43]. The cells were then fixed and permeabilized by using a BD cytofix/cytoperm plus kit and then stained for intracellular IFN-γ for 30 min at 4**°**C [44]. Immunophenotyping of lymphocytes was performed following red blood cell lysis on blood samples collected with heparin-coated syringes by heart puncture from S1P1 eGFP knock-in mice. Cells were then Fc blocked with anti-CD16/32 1:200 and stained with PE-conjugated rat anti-mouse CD3. Samples were then analyzed on a BD LSR II flow cytometer.

### Proliferation assay

Splenocytes were isolated from mice at day 8 following i.p. infection with 2.5 × 10^5^ PFU of the DM strain of mouse hepatitis virus (MHV-DM). Enriched populations of CD4+ and CD8+ T cells, isolated according to the manufacturer’s instructions (CD4 and CD8 Isolation kits, Miltenyi Biotec, Auburn, CA, USA), were labeled with the fluorescent dye, carboxyfluorescein diacetate succinimidyl ester (CFSE) (Life Technologies, Grand Island, NY, USA), at 2.5 μM final concentration. Then 1 × 10^6^ total cells per well were incubated with FTY720P 100 nM or vehicle and stimulated with 5 μM final peptide concentration of CD4+ T cell immunodominant epitope M133-147, CD8+ T cell immunodominant epitope S510-518, or non-specific OVA control, and cultured for 72 h at 37°C, 5% CO_2_ in complete media. Cells were then washed and the Fc receptor blocked with 1 × PBS containing 1% BSA and a 1:200 dilution of rat anti-mouse CD16/32 antibody (Pharmingen, San Jose, CA, USA). Next, cells were stained for surface antigens using APC-conjugated rat anti-mouse CD4 and CD8 (Pharmingen, San Jose, CA, USA), according to the viral peptide stimulation condition, for 45 min at 4°C. Cells were analyzed and the data assessed as described above.

### Cytotoxic T lymphocyte assay

Spleen-derived CD8+ T cells were analyzed for lytic activity at day 8 following i.p. infection of C57BL/6 mice with 2.5 × 10^5^ PFU of MHV-DM. A CD8+ T cell-enriched population of cells was obtained via negative selection through use of a magnetically labeled antibody specific for the CD8 antigen followed by passage over a magnetic column (Miltenyi Biotec, Auburn, CA, USA) [45]. The numbers of S510-518-specific CD8+ T cells were determined by tetramer staining and these cells were used as the effector population. RMA-S cells, a murine lymphoma cell line that presents viral peptides to cytotoxic T lymphocytes (CTL), were cultured at a density of 10,000 per well in flat-bottomed 96-well format tissue culture plate (Corning Life Sciences, Tewksbury, MA, USA) and pulsed overnight with 5 μM of the immunodominant CD8 peptide specific for MHV spike (S) glycoprotein, spanning amino acids 510 to 518 (S510-518, Bio-Synthesis, Lewisville, TX, USA). CD8 T-cells, exposed to either FTY720P (100 nM) or vehicle alone, were then plated with RMA-S cells at effector-to-target (E:T) ratios ranging from 20:1 to 2.5:1. Co-cultures were incubated for 4 h at 37°C in 5% CO_2_ at a final volume of 200 μl per well. The amounts of lactate dehydrogenase (LDH) released from lysed cells were determined using a CytoTox 96 Non-Radioactive Cytotoxicity Assay (Promega, Madison, WI, USA). The percentage of CTL-mediated lysis was determined as specified by the manufacturer’s protocols.

### Cytokine production

Spleen-derived CD4+ and CD8+ T cells from MHV-DM infected mice [39] were analyzed for cytokine secretion. CD4+ and CD8+ T cells were isolated as described above using an isolation kit according to the manufacturer’s instructions (Miltenyi Biotec, Auburn, CA, USA). Then 1 × 10^6^ T cells per well on a round bottom 96-well plate were incubated for 48 h at 37°C in 5% CO_2_ in the presence of FTY720P 100 nM or vehicle. Supernatants were then collected and an ELISA was performed for the following cytokines: IFN-γ and tumor necrosis factor alpha (TNF-α). Samples were run in triplicate in accordance with the manufacturer’s directions (R&D Systems, Minneapolis, MN, USA).

### Histology

Clinical severity was assessed using a previously described four-point scoring scale [16]. Spinal cords were isolated at defined time points and fixed overnight with 4% paraformaldehyde at 4°C. Spinal cords were separated into 12 coronal sections, cryoprotected in 20% sucrose and embedded in optimum cutting temperature (O.C.T) formulation (VWR, Radnor, PA, USA) [46]. Next 8-μm-thick coronal sections were cut and sections were stained with luxol fast blue (LFB). Areas of total white matter and demyelinated white matter were determined with Image J Software. Demyelination was scored as a percentage of total demyelination along the entire length of the spinal cord. An H&E stain was performed to determine the extent of inflammation. Spinal cord sections were scored using a four-point scale to assess neuroinflammation [16].

## Results

### FTY720 treatment of JHMV-infected mice increases clinical disease severity and impairs control of viral replication

**Figure 1:**
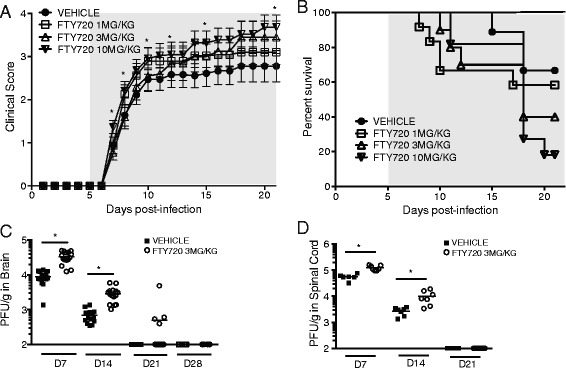
**FTY720 treatment increases clinical disease severity, mortality and affects viral clearance from the CNS. (A)** S1P1 eGFP knock-in mice C57BL/6 were infected i.c. with JHMV (150 PFU) and treated daily beginning at day 5 p.i. (shaded area) with either FTY720 (1, 3 and 10 mg/kg, *n* = 10 per group) or vehicle control by i.p. injection. Infected mice showed increased clinical disease severity at all FTY720 concentrations used with 10 mg/ml (*P < 0.05) having the greatest effect compared to vehicle-treated control mice. Data are presented as average ± standard error of the mean (SEM) and represent a minimum of two independent experiments with a minimum of five mice/group. **(B)** FTY720 treatment (beginning at day 5 p.i., shaded area) results in a dose-dependent increase in mortality compared to vehicle-treated mice. Data are representative of two independent experiments with a minimum of five mice/experimental group. Viral titers within the brain **(C)** and spinal cord **(D)** in JHMV-infected mice treated with either FTY720 (3 mg/kg) or vehicle (beginning at day 5 p.i.) were determined at days 7, 14 and 21 p.i. Data points represent individual mice and bars indicate averages. CNS viral titers represent two independent experiments. *P <0.05.

S1P1 eGFP knock-in mice C57BL/6 mice [36] were infected i.c. with JHMV (150 PFU) and subsequently treated with increasing concentrations (1, 3 or 10 mg/kg) of FTY720 via i.p. injection administered daily starting at day 5 p.i. Mice were scored daily until day 21 p.i. FTY720 treatment resulted in increased severity of clinical disease with the greatest effects occurring at 3 mg/kg and 10 mg/kg doses (*P* < 0.05) compared to vehicle-treated mice (Figure 1A). In accordance with clinical data, FTY720-treated mice exhibited increased mortality in a dose-dependent manner (Figure 1B). By day 21 p.i., <30% of mice treated with 10 mg/kg FTY720 survived while mice treated with either 3 mg/kg or 1 mg/kg exhibited 40% and approximately 60% survival, respectively (Figure 1B). Based on the morbidity and mortality data, 3 mg/kg FTY720 was used for subsequent *in vivo* studies. FTY720 treatment resulted in increased viral burden within the brain and spinal cord as determined by plaque titer at days 7 and 14 p.i.(*P* ≤ 0.05) compared to vehicle-treated control mice (Figure 1C,D). However, at later times p.i. the majority of mice treated with FTY720 had reduced viral titers below the level of detection (approximately 100 PFU/g) within the brain and spinal cord (Figure 1C,D). These findings indicated that the increase in mortality following administration of FTY720 correlated with impaired ability to control viral replication within the CNS and argues that either trafficking of virus-specific lymphocytes is impaired and/or anti-viral effector functions are negatively affected.

### T cell anti-viral effector function and FTY720 treatment

**Figure 2:**
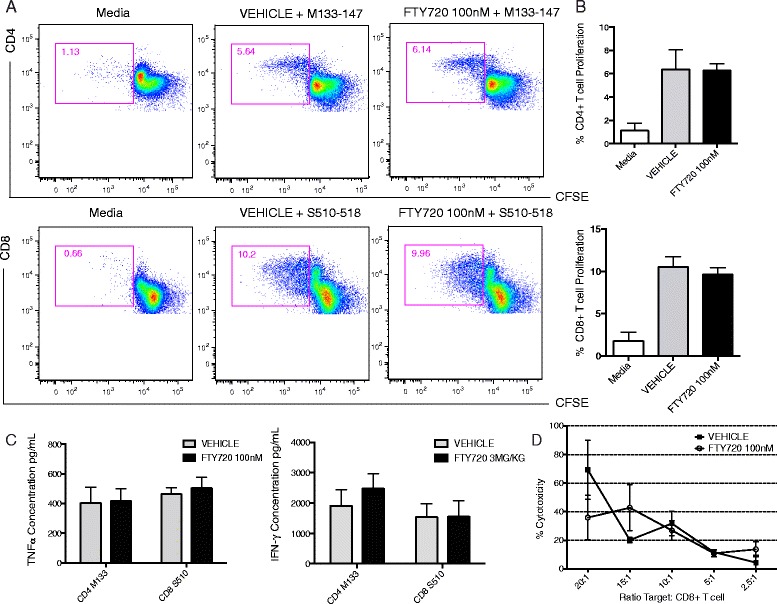
**FTY720 treatment does not impair T cell anti-viral effector function.** Representative flow data **(A)** showing proliferation (CFSE staining) of virus specific CD4+ and CD8+ T cells in response to treatment with FTY720P (100 nM). **(B)** Exposure to FTY720 does not affect proliferation compared to vehicle-treated controls. Data are presented as average + SEM and represent two independent experiments. **(C)** Cytokine production by CD4+ and CD8+ T cells obtained from JHMV-infected mice and subsequently stimulated with either matrix (M) glycoprotein 133–144 CD4 immunodominant epitope or spike (S) glycoprotein 510–518 CD8 immunodominant epitope and incubated with or without FTY720P (100 nM) for 48 h, at which point supernatants were collected and indicated cytokine levels determined by ELISA. **(D)** LDH assay showing target cell lysis (RMA-S cells were pulsed with 5 μM S510-518 peptide) by CD8+ T cells pre-incubated with FTY720P 100 nM or vehicle. LDH release was determined after 4 h incubation at 37°C. Data are representative of two independent experiments and presented as average ± SEM.

T cell responses, including proliferation, secretion of IFN-γ and CTL activity, are critical in controlling JHMV replication within the CNS [47–53]. FTY720P treatment (100 nM) had no appreciable effect on dampening proliferation of either CD8+ T cells specific for the immunodominant epitope for the spike (S) glycoprotein spanning amino acid residues 510–518 (S510-518) [42] or CD4+ T cells recognizing the matrix (M) glycoprotein peptide 133–147 (M133-147) [43] (Figure 2A,B). Lymphocytes were isolated from spleens of JHMV-DM infected mice day 8 p.i., pulsed with either M133-147 or S510-518 peptides and treated with FTY720P (100 nM) to determine if cytokine secretion was affected. FTY720 treatment did not affect secretion of either IFN-γ or TNF-α compared to vehicle-treated cultures (Figure 2C). Finally, FTY720 treatment of CD8+ T cells did not affect lytic activity compared to controls (Figure 2D). These findings argue that FTY720 treatment does not dampen anti-viral T cell effector functions.

### FTY720 treatment impairs T cell trafficking into the CNS

**Figure 3:**
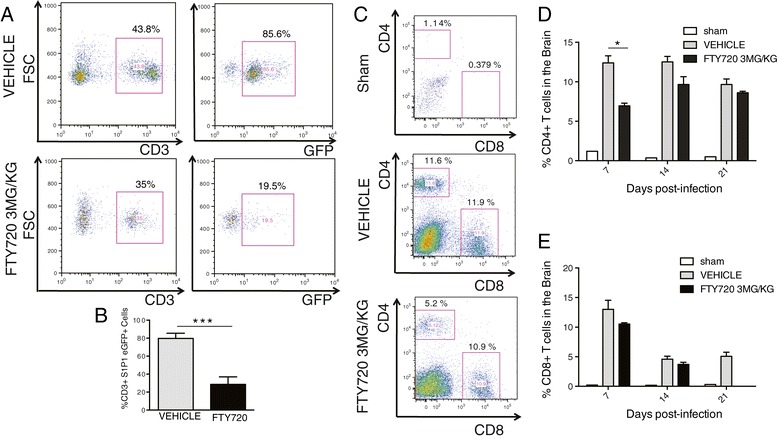
**FTY720 treatment reduces T cell infiltration into the CNS of JHMV-infected mice.** S1P1 eGFP mice were i.c. infected with JHMV (150 PFU) and treated daily with FTY720 (3 mg/kg) starting on day 5 p.i. **(A)** Representative flow data and **(B)** frequencies of CD3+ T cells showing reduced expression of S1P1 (as determined by eGFP expression) in blood at day 7 p.i. **(C)** Representative flow data showing CD4+ and CD8+ T cell accumulation within the CNS of sham-infected and JHMV-infected eGFP S1P1 mice treated daily with vehicle or FTY720 (3 mg/kg) starting at day 5 p.i. Frequencies of CD4+ **(D)** and CD8+ T cells **(E)** present within the brains of either vehicle- or FTY720-treated infected mice as well as sham-infected controls at defined times p.i. Data in panels B, D and E are presented as average + SEM and represent three independent experiments with a minimum of five mice/group. *P <0.05, *** *P* <0.001. FSC, forward scatter.

We next determined if FTY720 affected S1P1 expression on circulating lymphocytes in JHMV-infected mice. Administration of FTY720 reduced S1P1 on circulating CD3+ lymphocytes (*P* < 0.001) compared to vehicle-treated controls at day 7 p.i. (Figure 3A,B). Examination of T cell infiltration into the CNS of FTY720-treated mice infected with virus indicated reduced frequency of CD4+ T cells (*P* < 0.05) at day 7 p.i. although CD4+ T cell trafficking was not affected at days 14 and 21 p.i (Figure 3C,D). Further, FTY720 treatment did not affect CD8+ T cell infiltration into the CNS at days 7, 14 and 21 p.i. (Figure 3C,E).

**Figure 4:**
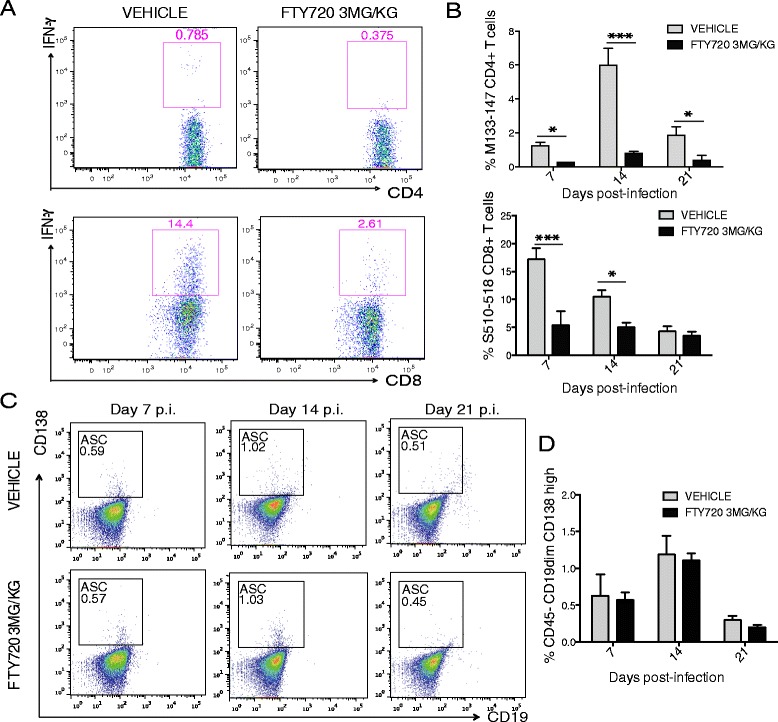
**FTY720 treatment affects virus-specific T cell infiltration into the CNS.** S1P1 eGFP mice were i.c. infected with JHMV (150 PFU) and treated daily with FTY720 (3 mg/kg) starting on day 5 p.i. **(A)** Representative flow staining showing intracellular IFN-γ staining by CNS infiltrating T cells following exposure to CD4+ T cell epitope (M133-144) and CD8+ T cell epitope (S510-518) at day 7 p.i. in mice treated with either FTY720 (3 mg/kg) or vehicle control. **(B)** Reduced frequency of virus-specific IFN-γ producing CD4+ and CD8+ T cells in the CNS following FTY720 treatment at defined times p.i. **(C, D)** Similar frequencies of infiltrating ASCs (CD45^−^CD19^low^ CD138^+^) in the brains of vehicle- and FTY720-treated mice. Data in B and D are presented as average ± SEM and represent a minimum of two independent experiments with at least five mice/experimental group. *P <0.05, *** *P* <0.001.

Infiltration of virus-specific CD4+ and CD8+ T cells, as determined by intracellular IFN-γ staining in response to treatment with immunodominant CD4+ and CD8+ viral epitopes [42,43], was diminished at days 7 (*P* < 0.01) and 14 (*P* < 0.05) following FTY720 treatment in comparison to vehicle-treated mice (Figure 4A,B). By day 21 p.i., infiltration of virus-specific CD4+ T cells, but not virus-specific CD8+ T cells, was also reduced (*P* < 0.05) in FTY720-treated mice compared to control animals (Figure 4B). Infiltration of antibody secreting cells (ASCs) (CD45^−^CD19^low^ CD138+) into the CNS of JHMV-infected mice was not reduced at either days 7, 14 or 21 p.i. following FTY720 treatment compared to control mice (Figure 4C,D). These findings indicate that administration of FTY720 negatively impacts recruitment of T lymphocytes into the CNS in response to JHMV infection.

### FTY720 restricts lymphocyte egress from draining cervical lymph nodes

**Figure 5:**
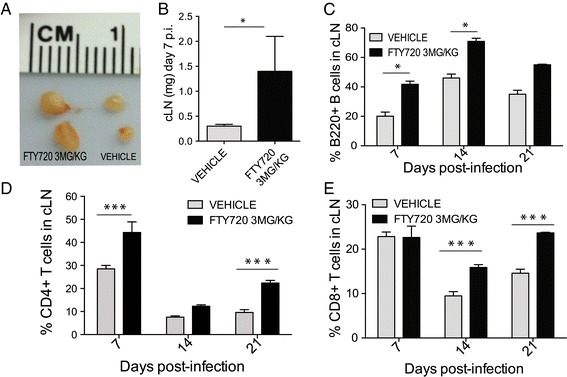
**Increased lymphocyte retention in dCLNs following FTY720 treatment of JHMV-infected mice.** S1P1 eGFP mice were i.c. infected with JHMV (150 PFU) and treated daily with FTY720 (3 mg/kg) beginning on day 5 p.i. On days 7, 14 and 21 p.i., dCLNs were isolated to examine size, weight and immunophenotype lymphocyte population by flow cytometry. **(A)** Representative image depicting the increase in size of dCLNs obtained from FTY720-treated mice compared to vehicle control-treated mice at day 7 p.i. **(B)** Average weight of dCLNs increased in response to FTY720 treatment compared to vehicle (*P* < 0.05). Treatment with FTY720 resulted in increased retention of B220+ B cells **(C)**, CD4+ T cells **(D)** and CD8+ T cells **(E)** at defined times p.i. Data in panels B to E represent average ± SEM obtained from two or three independent experiments with a minimum of four mice/group. *P <0.05, *** *P* <0.001. cLN, cervical lymph node. dCLN, draining cervical lymph node.

As an S1P1 functional antagonist, FTY720 disrupts the S1P gradient in lymph nodes thereby trapping lymphocytes [4,5]. To establish if this occurs during ongoing neuroinflammation in response to infection with neurotropic JHMV, draining cervical lymph nodes (dCLNs) were examined at defined times p.i. following treatment with FTY720. Administration of FTY720 revealed an increase in size and weight of dCLNs in FTY720-treated mice compared to control mice at day 7 p.i. (Figure 5A,B). Immunophenotyping lymphocytes in dCLNs by flow cytometry revealed an increased frequency of B220+ B cells (Figure 5C), CD4+ T cells (Figure 5D) and CD8+ T cells (Figure 5E) in mice treated with FTY720 compared to controls, indicating increased retention of lymphocytes in lymphatic tissue in response to S1P antagonism.

### FTY720 diminishes the severity of demyelination in JHMV-infected mice

**Figure 6:**
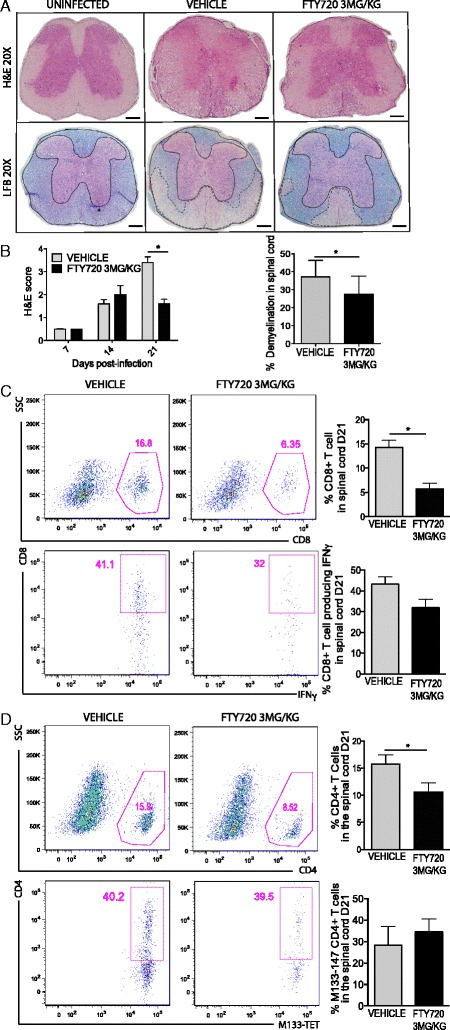
**FTY720 treatment reduces the severity of JHMV-induced demyelination. (A)** Representative LFB and H&E-stained spinal cord images showing an overall reduction in the severity of inflammation and demyelination within white matter tracts (dashed lines) in JHMV-infected eGFP S1P1 mice treated with FTY720 (3 mg/kg) compared to vehicle control-treated mice at day 21 p.i. **(B)** FTY720 treatment results in reduced neuroinflammation (*P* < 0.05) and demyelination (*P* < 0.05) compared to mice treated with the vehicle control. Flow analysis of spinal cords demonstrates reduced entry of both CD8+ **(C)** and CD4+ T cells **(D)**, while infiltration of virus-specific T cells was not affected. Data in panel B represent two independent experiments with a minimum of ten mice/group. Data in panels C and D are presented as average + SEM and represent two independent experiments with a minimum of five mice/group. *P <0.05. The scale bar in panel A represents 200 μm. SSC, side scatter.

To investigate the potential effect of FTY720 on spinal cord neuroinflammation and demyelination in JHMV-infected mice, an evaluation of the severity of white matter damage was performed at day 21 p.i. Spinal cord inflammation was reduced (*P* < 0.05) within spinal cords at day 21 p.i. as assessed by H&E staining (Figure 6A,B). Moreover, LFB staining revealed a significant (*P* < 0.05) reduction in demyelination in response to FTY720 treatment compared to control mice (Figure 6A,B). Immunophenotyping of infiltrating lymphocytes in the spinal cord day 21 p.i. revealed a decrease in CD8+ T cell as well as CD4+ T cell percentages (*P* <0.05) in FTY720-treated mice compared to vehicle-treated mice (Figure 6C,D). This suggests that reduction in the severity of demyelination following FTY720 treatment is the result of a decrease in infiltration of inflammatory cells into the spinal cord.

## Discussion

FTY720/fingolimod was the first oral treatment approved by the FDA for relapsing forms of MS [10,54,55]. Numerous clinical trials highlighted FTY720 efficacy as demonstrated by benefits for relapses and magnetic resonance imaging (MRI) lesions [9]. In addition, disability progression was impacted and there was a reduction in brain volume loss in MS patients in response to treatment [56,57]. Although the mechanisms by which FTY720 exerts a protective effect are not defined, it is generally accepted that the main mode of action is via an immunomodulatory effect by restricting lymphocyte circulation from lymph nodes to the CNS. Dampened accumulation of activated lymphocytes in the CNS in response to FTY720 treatment most likely accounts for reduced MRI lesion activity and this is supported in preclinical animal studies using EAE [14,15,58]. Whether the reduction in brain volume loss is also dependent upon reduced neuroinflammation or a direct neuroprotective effect has not been determined. Recent evidence argues that FTY720 exerts a neuroprotective effect as animals in which the receptor S1P1 is selectively ablated on astrocytes are resistant to the protective effects of FTY720 treatment following induction of EAE, although S1P1 remains expressed on circulating lymphocytes [12,59]. However, more recent studies by Cahalan *et al*. indicate that S1P1 antagonism reverses EAE without acting on S1P1 expressed within the CNS, supporting the notion that restricting lymphocyte egress from lymphatic tissue is sufficient to diminish disease severity [60]. Moreover, the maintenance of egress inhibition is not required for efficacy in EAE if brain levels of agonist are maintained in the steady state. Full efficacy is achieved with inhibition of egress for only 30% of the 24-h dosing interval with complete recovery of circulating lymphocytes. Direct effects within the CNS were demonstrated for neurons, astrocytes and the blood–brain-barrier, and on the inhibition of migration of lymphocytes from perivascular cuffs into the parenchyma [61].

We chose a model of viral-induced neurologic disease to determine if FTY720 treatment affects host defense and disease progression. Our rationale for using the JHMV model of acute encephalomyelitis and demyelination to assess the therapeutic benefit of FTY720 is based on the fact that the overwhelming majority of preclinical animal models examining the mode of action for FTY720 is derived from EAE, yet how this drug affects models of viral-induced CNS disease are not well characterized. In addition, viral infections have long been thought to have a role in either initiating or contributing to relapse in MS patients [28–34,62]. How treatment with FTY720 influences outcomes in response to viral infection is highlighted by recent clinical studies detailing the emergence of herpes zoster and associated neurologic complications in MS patients during FTY720 treatment [63,64]. These findings suggest immunosuppression may arise in response to FTY720 treatment resulting in re-emergence of persistent viruses. However, administration of FTY720 to mice infected with lymphocytic choriomeningitis did not ameliorate persistence, indicating that the outcome may be dictated, in part, by the virus and sites of infection [65]. Related to these observations are studies demonstrating that treatment with FTY720 or other S1P1 agonists dramatically affects cytokine production and disease outcome in mice infected with influenza virus, indicating the immunomodulatory effects of such treatment [66]. With regards to viral models of demyelination, Pachner and colleagues [67] demonstrated that administration of FTY720 had no effect on clinical disease progression or viral load within the CNS using Theiler’s murine encephalomyelitis virus model of demyelination. These findings are in contrast with findings using EAE, in which FTY720 treatment reduced clinical disease severity accompanied by limited infiltration of immune cells into the CNS [13–15,68,69].

Our findings show that FTY720 treatment for JHMV-infected mice increased clinical disease severity as well as mortality. Importantly, these findings correlate with impaired ability to control viral replication within the CNS. The muted host defense resulting from S1P receptor antagonism was not the result of dampened anti-viral T cell effector responses, e.g. proliferation, cytokine secretion or CTL activity, but rather an inability of lymphocytes to migrate and accumulate within the CNS effectively. Indeed, administration of FTY720 increased retention of T and B lymphocytes within the dCLNs, consistent with earlier reports that blocking S1P receptors disrupts lymphocyte egress from secondary lymphatic tissue [70,71]. Although viral titers were elevated within the CNS of FTY720-treated mice, surviving mice were able to reduce the amount of virus below the level of detection and this lasted to day 28 p.i., arguing that viral recrudescence does not occur in this model.

Administration of FTY720 either prophylactically or therapeutically in models of EAE results in reduced lymphocyte penetration into the CNS, which is associated with a reduction in the severity of demyelination [14,15,58]. Similarly, our results show that the effects of FTY720 treatment on CNS inflammation in JHMV-infected mice correlates with a reduction in the severity of spinal cord demyelination. Examination of the posterior funiculus and lateral white matter columns of FTY720-treated mice compared to controls showed an overall reduction in lesion size, demonstrating that in addition to reducing myelin damage in EAE, FTY720 is also effective in limiting the spread of demyelination in a viral model of MS. FTY720 has also been shown to prevent axonal damage in EAE [58]. FTY720 in combination with other drugs in EAE or cerebellar slice cultures has been shown to augment remyelination, supporting a regenerative potential [72,73]. These findings support that FTY720 may act directly upon resident cells of the CNS promoting protection and repair. This is supported by elegant studies from Chun and colleagues [12] that showed attenuation in EAE and a loss of FTY720 efficacy in conditional null mouse mutants lacking S1P1 in astrocytes. These findings highlight that FTY720-mediated protection in EAE occurs via a nonimmunological mechanism and suggest that targeting S1P signaling within the CNS may be relevant for recovery for both EAE and MS patients. Whether extensive axonal sparing and/or remyelination occurs following FTY720 administration to JHMV-infected mice is not known at this time and is an area of ongoing work.

## Conclusions

In this study we demonstrate that FTY720 mutes effective anti-viral immune responses by preventing migration and accumulation of virus-specific T cells within the CNS during acute viral-induced encephalomyelitis. FTY720 treatment reduces the severity of neuroinflammatory-mediated demyelination by limiting T cell egress from lymph nodes thereby reducing lymphocyte infiltration into the CNS. FTY720 did not alter anti-viral effects of T cells, e.g. cytokine secretion or cytolytic activity.

## Abbreviations

ASC: antibody secreting cell
BSA: bovine serum albumin
CFSE: carboxyfluorescein succinimidyl ester
CNS: central nervous system
CTL: cytotoxic T lymphocyte
dCLN: draining cervical lymph node
EAE: experimental autoimmune encephalomyelitis
ELISA: enzyme-linked immunosorbent assay
FDA: Food and Drug Administration
FTY720P: FTY720-phosphate
H&E: hematoxylin and eosin
i.c.: intra-cerebral
IFN-γ: interferon-gamma
i.p.: intra-peritoneal
JHMV: JHM strain of mouse hepatitis virus
LDH: lactate dehydrogenase
LFB: luxol fast blue
MHV: mouse hepatitis virus
MRI: magnetic resonance imaging
MS: multiple sclerosis
PFU: plaque forming units
p.i.: post-infection
S1P: sphingosine-1-phosphate
SEM: standard error of the mean
TNF-α: tumor necrosis factor alpha
